# Whole-Body MRI Screening for Carriers of Germline TP53 Mutations—A Systematic Review and Meta-Analysis

**DOI:** 10.3390/jcm13051223

**Published:** 2024-02-21

**Authors:** Hugo C. Temperley, Niall J. O’Sullivan, Benjamin M. Mac Curtain, Wanyang Qian, Tatiana S. Temperley, Alannah Murray, Alison Corr, Ian Brennan, David Gallagher, James F. Meaney, Michael E. Kelly

**Affiliations:** 1 Department of Radiology, St. James’s Hospital, D08 NHY1 Dublin, Ireland; 2 Department of Surgery, St. James’s Hospital, D08 NHY1 Dublin, Ireland; 3 Department of Urology, St. Vincent’s University Hospital, D04 T6F4 Dublin, Ireland; 4 St John of God Midland Hospital, Midland, WA 6056, Australia; 5 School of Medicine, University of Limerick, V94 T9PX Limerick, Ireland; 6 Department of Genetics, St. James’s Hospital, D08 NHY1 Dublin, Ireland

**Keywords:** whole-body MRI, cancer screening, TP53 mutations, Li-Fraumeni syndrome

## Abstract

Purpose: This systematic review evaluated whole-body MRI (WB-MRI) as a cancer screening tool for individuals carrying germline TP53 mutations, a population known to be at a significantly elevated risk of malignancy. The primary objective is to assess the diagnostic performance of WB-MRI in detecting cancer in this cohort. Methods: PubMed, MEDLINE, EMBASE and the Cochrane Central Registry of Controlled Trials were searched until 18 August 2023. Eligible studies were selected based on predefined inclusion criteria. The data extracted included information on study characteristics, patient demographics, and the WB-MRI diagnostic performance. Results: This systematic review identified eight eligible studies, comprising 506 TP53 mutation carriers. The mean age was 34.6 ± 16.3 (range 1–74) years. In total, 321/506 (63.4%) of the patients were female and 185/506 (36.6%) were male. In addition, 267/506 (52.8%) had a previous oncological diagnosis. Thirty-six new cancers were diagnosed with WB-MRI (36/506 (7.1%)). The overall pooled proportion of cancer detected on MRI was 7% (95% confidence interval 5–10). In total, 44 new lesions were picked up, as multiple lesions were found in some patients. Conclusion: WB-MRI is an effective cancer screening tool for TP53 mutation carriers. While these findings suggest the potential for WB-MRI to contribute to early cancer detection in this high-risk population, further research and the standardisation of protocols internationally are warranted to optimise its clinical utility.

## 1. Introduction

Germline TP53 mutations cause Li-Fraumeni syndrome (LFS), a rare genetically inherited autosomal dominant condition that significantly elevates the lifetime risk of developing multiple types of cancers [1,2,3,4], including breast, brain and/or sarcomatous lesions [5]. The reported rates of cancer development are 22% by the age of 5, 41% by age 18 and almost 100% by age 70 [6,7]. Unlike other hereditary cancer syndromes, in LFS, the universal radiological guidelines for cancer screening are not as clear [8]. Despite this, emerging data suggest that surveillance may offer benefits [8,9,10]. Furthermore, surveillance guidelines for individuals carrying disease-causing TP53 mutations have recently been outlined through the collaborative efforts of an international consortium led by Canadian and US teams [8,11]. These guidelines recommend that carriers initiate annual WBMRI and annual brain MRI from the first year of life [8].

Several global studies, mostly conducted without gadolinium-based contrast agents (GBCAs), have validated the effectiveness of WBMRI, showing an overall estimated detection rate of 7% for new and localised primary cancers during the initial screen [8,12,13]. Given the potential long-term retention of GBCAs in various organs, it is recommended that caution is exercised when administering multiple GBCAs to germline TP53 variant carriers. Furthermore, only macrocyclic GBCAs, known for their apparently lower retention in the body [14], should be utilised.

Research is ongoing to establish effective strategies for the early detection and surveillance of cancer in TP53 mutation carriers [15,16]. Traditional cancer screening paradigms have often proven inadequate for this high-risk population, necessitating a more nuanced and comprehensive approach [17,18]. WB-MRI, a non-invasive imaging modality, has emerged as a promising contender in this pursuit [19,20,21]. WB-MRI can scrutinise soft tissues and organs throughout the body without the need for ionising radiation [22]. This non-radiating characteristic makes it an attractive option for repeated surveillance in individuals predisposed to cancer, thus mitigating the cumulative risks associated with radiation exposure from computed tomography (CT) scans [23,24].

The deployment of WB-MRI as a primary cancer screening tool for LFS poses several clinical, logistical, and financial implications [13]. Its efficacy in reliably detecting cancers at an early, treatable stage in this unique population remains controversial [10,25]. This systematic review aims to synthesise the existing body of literature regarding the utility of WB-MRI as a cancer screening modality for individuals with LFS.

## 2. Methods

### 2.1. Registration and Search Strategy

**Figure 1:**
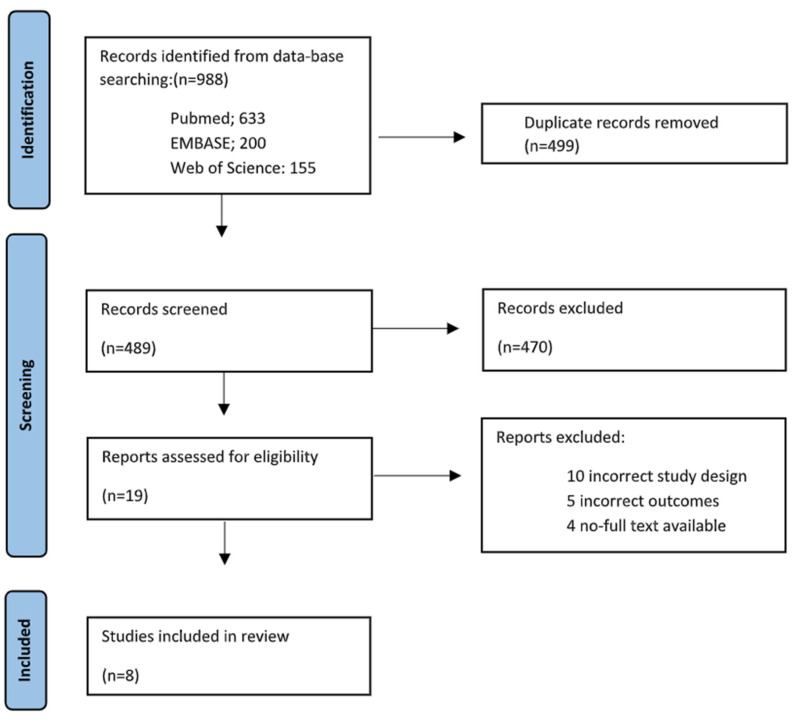
PRISMA flowchart outlining the systematic search process.

Our search was conducted in line with the most recent Preferred Reporting Items for Systematic Reviews and Meta-Analyses (PRISMA) recommendations [26]. Our study protocol was prospectively registered with PROSPERO (CRD42023400797). We conducted a search using PubMed, MEDLINE (Ovid), EMBASE and the Cochrane Central Register of Controlled Trials using a search strategy undertaken on 18 August 2023. The search pathway is illustrated in the PRISMA diagram in Figure 1. The grey literature was also searched for any relevant studies. The systematic search process with detailed search terms are outlined in Supplementary Material S1. Due to heterogeneity and the descriptive nature of the results that are presented, a narrative summary of findings is presented.

### 2.2. Inclusion/Exclusion Criteria

#### 2.2.1. Inclusion Criteria

For analytical inclusion, studies had to meet the following criteria:

(a) Report on patients with LFS who underwent WB-MRI radiological oncological screening.
(b) Report on cancers picked up on WB-MRI.
(c) Report with a well-defined research methodology.

#### 2.2.2. Exclusion Criteria

Studies were excluded from the analysis if the following were true:

(a) Patients did not have a diagnosis of LFS.
(b) Imaging was performed by another radiological modality, other than WB-MRI.
(c) Outcomes of interest were not reported.
(d) The methodology was not clearly reported.

### 2.3. Identification of Studies and Outcomes of Interest

Studies that satisfied the inclusion and exclusion criteria were included in our review. The information extracted was based on the PICO framework (Population,

Intervention, Comparator, and Outcomes) [27]. The following PICO elements were used as the basis for selecting studies:

I. Population: LFS patients
II. Intervention: LFS patient undergoing WB-MRI for cancer screening
III. Comparison: asymptomatic non-LFS patients undergoing WB-MRI
IV. Outcome:

Primary outcome: incidence of new cancer diagnosis on WB-MRI.

### 2.4. Study Selection, Data Extraction and Critical Appraisal

A database was created using the reference managing software EndNote X9 TM Number 21. The abstracts of articles yielded from the search were reviewed by two independent authors (HCT and NOS) based on the inclusion and exclusion criteria detailed above. Following the removal of duplicate articles, discrepancies in judgment about the relevance of articles were resolved via an open discussion between the authors and an independent third reviewer (MK). An article was excluded from the review when the three reviewers came to an agreement. Full texts of the shortlisted articles were obtained and further evaluated to ensure that they met our inclusion criteria. The references of the shortlisted articles were then searched to identify other relevant studies that may have been missed through the initial search of online databases. Data were extracted by two reviewers independently from the articles that met the inclusion criteria based on a full-text review. In order to extract and store the data efficiently, the Cochrane Collaboration screening and data extraction tool, Covidence version 2.0, was used [28]. 

### 2.5. Statistical Analysis

A proportional meta-analysis was performed [29]. Statistical analysis was run using Stata 17 [30]. Proportions were pooled using the “metaprop” function within Stata; 95% confidence intervals were employed and *p* ≤ 0.05 was considered statistically significant. Heterogeny was reported using I^2^, with >−50% considered significant [31].

### 2.6. Risk of Bias

The potential biases were assessed using the Newcastle–Ottawa scale (HT) risk of bias tool and the results were tabulated [32]. This assessment tool grades each study as being ‘satisfactory’ or ‘unsatisfactory’ across various categories. We assigned stars to evaluate the study quality: 7 stars—“very good”, 5–6 stars “good”, 3–4 stars “satisfactory” and 0–2 stars “unsatisfactory”. The critical appraisal was completed by two reviewers independently (HT and NOS), where once again a third reviewer (MK) was asked to arbitrate when there were discrepancies in opinion (See Supplementary Material S2).

## 3. Results

### 3.1. Search Results

In total, 988 articles were identified and 499 duplicate articles were excluded. Thereafter, the study titles and abstracts were screened, resulting in 19 studies being eligible for full-text review. Of these, eight studies met the eligibility criteria and were included [12,13,20,25,33,34,35,36]. The PRISMA flow chart is illustrated in Figure 1. Overall, six studies were prospective cohort studies [12,20,25,34,35,36], and the remaining two studies were retrospective cohort studies [33,34]. Only one study used a control group [33]. The primary outcome was the identification of lesions suspicious for malignancy in asymptomatic TP53 carriers in seven studies, whilst Kagami et al. defined the primary outcome as radiological findings leading to follow-up imaging or intervention in asymptomatic TP53 carriers [34].

### 3.2. Patient Characteristics

Overall, 550 patients who had body MRI were included; of these, 506/550 (92%) of the patients had a known diagnosis of LFS, the remaining 44 being from the control group in the study by Saya et al. [33] Five studies examined adults and children [13,25,33,34,35], two studies examined children only [20,36], and one study examined adults only [12]. The mean age was 34.6 ± 16.3 (range 1–74) years. In total, 321/506 (63.4%) of the patients were female and 185/506 (36.6%) were male. Overall, 267/506 (52.8%) had a previous cancer diagnosis. See Table 1 for characteristics of the trials and populations.

### 3.3. MRI Protocols/Acquisition Parameters

All eight studies employed MRI as the only imaging modality for screening. Overall, four studies reported on scanning time; however, only Mai et al. reported a mean time of 45 min [35], whilst the other three studies reported a scanning time range (25–150 min) [20,25,36]. Five of the eight studies utilised an identical WB-MRI protocol in all subjects [12,13,25,33,36]. In the six studies that reported it, the MRIs were reviewed by at least two or more experienced radiologists. Only two studies employed gadolinium-based contrast imaging [13,35]. The full acquisition parameters are illustrated in Table 2.

### 3.4. Incidence of New Cancer

**Figure 2:**
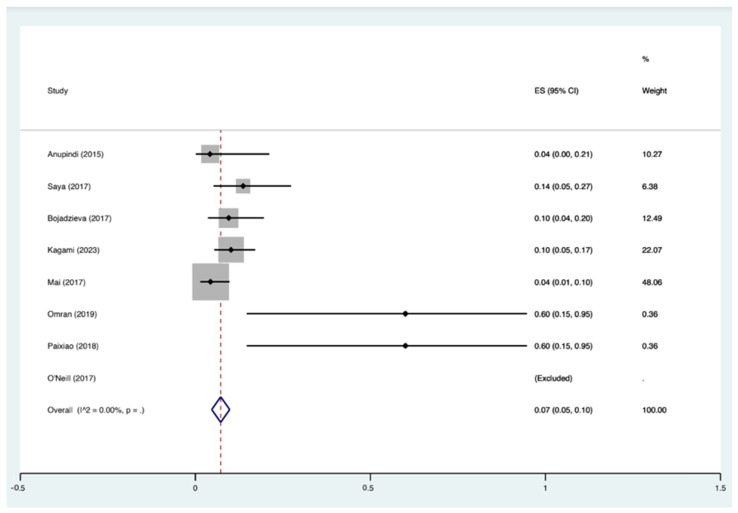
Overall pooled proportion of cancer detected on MRI. ES: Effect Sizes [12,13,20,25,33,34,35,36].

Overall, 44 new lesions were identified in 36 patients with WB-MRI (36/506 (7.1%)) and subsequently confirmed as cancer. The overall pooled proportion of cancer detected on MRI was 7% (95% CI 5–10) (Figure 2). The distribution of cancer diagnosis by location was as follows: 16 abdominal, 10 chest, 4 brain, 4 neck, 4 pelvis, 3 lower limb, 2 upper limb and 1 breast. Five studies specified whether the cancer was identified on the initial or follow-up WB-MRI scan [12,20,25,33,34]. Nine malignant lesions (in nine patients) that were not present on the initial scan were diagnosed during follow-up WB-MRI. The most common cancers were renal cell carcinoma, osteosarcoma, colorectal adenocarcinoma and astrocytoma. The confirmation of cancer in all studies was performed through a combination of further imaging and subsequent tissue specimen for histological diagnosis. See Table 3 for lesion diagnosis by body distribution and Table 4 for a descriptive overview of the types of cancer.

### 3.5. Risk of Bias

One study was ‘very good’, seven studies were ‘good’, zero studies were ‘satisfactory’ and zero studies were ’unsatisfactory’. Supplementary Material S2 summarises the results of our risk of bias assessment and individual breakdown of included studies.

## 4. Discussion

The management of individuals with germline TP53 mutations presents a unique challenge to screening services due to their significant risk of developing a wide spectrum of cancers over the course of their life [15]. Conventional cancer screening modalities are limited in detecting the full range of malignancies associated with TP53 mutations [35,37,38]. Whole-body MRI (WB-MRI) has emerged as a potential comprehensive tool in this context, offering a comprehensive, non-ionising radiation approach to screening for multiple cancers simultaneously [35].

Our review identified eight studies encompassing 506 TP53 variant carriers who underwent WB-MRI. These studies collectively revealed several critical insights into the potential of WB-MRI in early cancer detection for high-risk individuals and detected cancer in 7% (95% CI 5–10%). This figure emphasises that WB-MRI can detect asymptomatic cancers in TP53 variant carriers before reaching a more advanced stage. One of the strengths of WB-MRI lies in its ability to identify multiple lesions in a single scan. In our review, 44 new lesions were detected, underlining the comprehensiveness of this imaging approach. For individuals with TP53 mutations who are at risk of developing cancers in various organs, WB-MRI’s capacity to visualise multiple body regions simultaneously is a valuable asset. This advantage improved the efficiency of time while also reducing the burden of multiple screening tests, which TP53 mutation carriers are often subjected to.

While the findings of our review are encouraging, several important considerations should guide the future implementation of WB-MRI in clinical practice for TP53 mutation carriers. One key aspect is the need for the standardisation of protocols. Although surveillance protocols have been reported by several international consortiums [8,11,39], currently, there is no universally accepted WB-MRI protocol for LFS screening, and variations in scanning parameters and interpretation criteria exist [40,41]. Establishing standardised guidelines for image acquisition and interpretation will be crucial to ensure consistent and reliable results across different healthcare settings. Another critical point of discussion is the balance between the benefits and challenges of WB-MRI. One challenge highlighted in our review is the interpretation of MRI findings. The detection of incidental lesions can lead to unnecessary patient anxiety and invasive follow-up procedures, emphasising the importance of specialised centres employing the expertise of specialised radiologists experienced in WB-MRI screening [10,19]. Additionally, the cost and availability of WB-MRI should be considered, as these factors may limit its widespread adoption, particularly in resource-constrained healthcare systems [42,43,44]. In our systematic review, none of the studies examined the cost-effectiveness of WB-MRI in this cohort.

Integrating WB-MRI into a personalised screening plan, alongside other imaging modalities and clinical assessments, can provide a more comprehensive and targeted approach to cancer surveillance [8]. It is also worth noting that the 7.3% rate of new cancer diagnoses in our review suggests that a significant proportion of TP53 mutation carriers are developing cancers that may have been missed by conventional screening methods. This reinforces the need for improved surveillance strategies for this vulnerable population.

In conclusion, a systematic review of the literature provides compelling evidence for the use of WB-MRI as an effective cancer screening tool for TP53 mutation carriers and its ability to detect cancers in multiple organs in a single scan setting. However, the implementation of WB-MRI in clinical practice requires further validation, and in particular, simple and reproductive standardised protocols and cost-effectiveness analyses. The findings emphasise the importance of individualised risk assessment and highlights the role of WB-MRI in early cancer detection, which may ultimately improve the outcomes of TP53 mutation carriers.

## Figures and Tables

**Table 1:** Characteristics of the trials included.

Author	Year	Study Population	Country	Journal	Study Method	No. Patients	Age (Mean ± SD, Range)	Sex (M:F)	Previous Cancer	Primary Outcome
Anupindi [20]	2015	Children diagnosed with either Li-Fraumeni syndrome (LFS), hereditary paraganglioma-pheochromocytoma syndrome, or rhabdoid tumour syndrome	USA	American Journal of Roentgenology	Retrospective cohort	24	10.7 (± 4.6, 2.1–18.2)	6:18	7/24 (29%)	Identification of lesions suspicious for malignancy in asymptomatic TP53 pv carriers
Saya [33]	2017	TP53 pathogenic variant carriers, and age- matched (±5 years) and sex-matched population controls.	UK	Familial Cancer	Prospective cohort	88 (44 carrier and 44 control)	Carrier 38.1 (±11.3, 19–58) Control 39.4 (±10.7, 22–59)	Carrier 27:17, Control 27:17	18/44 (41%)	Identification of lesions suspicious for malignancy in asymptomatic TP53 pv carriers
Bojadzieva [13]	2017	Adults and children with Li-Fraumeni Syndrome	USA	Familial Cancer	Prospective cohort	63 (49 adults, 14 children). 53 underwent baseline WB-MRI, 35 underwent brain MRI only.	N/a	18:45	43 (68%)	Identification of lesions suspicious for malignancy in asymptomatic TP53 pv carriers
Mai [7]	2017	Patients with Li-Fraumeni syndrome aged 3 years or older at time of baseline screening and who had not received active cancer therapy at least 6 months prior to screening.	USA	JAMA Oncology	Prospective cohort	116	38.5 (±17.9, 3–65)	40:17	71/116 (61.2%)	Identification of lesions suspicious for malignancy in asymptomatic TP53 pv carriers
O’Neill [36]	2017	Paediatric patients with TP53 germline mutation, prior cancer patients in stable remission for at least 6 months post treatment	USA	Paediatric blood cancer	Prospective cohort	22	9.5 (±4.0, 1–15)	10:12	5/22 (33%)	Identification of lesions suspicious for malignancy in asymptomatic TP53 pv carriers
Paixiao [25]	2018	Patients with Li-Fraumeni syndrome (TP53 germline mutation) with no current disease	Brazil	Cancer Imaging	Prospective cohort	59	38 ± 11.1 (2–71)	24:35	27/59 (45%)	Identification of lesions suspicious for malignancy in asymptomatic TP53 pv carriers
Omran [12]	2019	Adult patients recruited by SWEP53 study, with verified likely pathogenic (class 4) or pathogenic (class 5) TP 53 variants	Sweden	Cancer	Prospective cohort	61	40 (±7.9, 18–74)	21:39	32/61 (52%)	Identification of lesions suspicious for malignancy in asymptomatic TP53 pv carriers
Kagami [34]	2023	Adults and children with Li-Fraumeni Syndrome	USA	American Association for Cancer Research	Retrospective cohort	118 (68 adult, 50 children)	N/a	41:18	64/118 (54%)—total 50/68 (74%)—adult 14/50 (28%)—paediatric	Radiological findings leading to follow-up imaging or intervention in asymptomatic TP53 pv carriers

**Table 2:** Characteristics of the MRI protocols/acquisition parameters.

Author	Scanning Time (minutes)	Identical WB-MRI Protocol in All Subjects (Y/N)	MRI Interpreter (Number of Rads/Level/Experience)	Phase	Model	Field Strength	FOV	TR/TE (ms)	ST (mm)	Matrix	Contrast
Anupindi [20]	39–150 min (range)	No	Two board certified radiologists with at least 5 year’s paediatric full body MRI experience	coronal STIR, coronal T1-weighted, axial STIR, and axial T2-weighted fat-suppressed sequences (protocol changed during study)	Avanto or Symphony, Siemens Healthcare (n = 30/50 scans) Skyra, Verio, or Trio, Siemens Health-care) (20/50 scans)	1.5 T (30/50 scans), 3T (20/50 Scans)	Variable based on patient size	1.5 T Coronal STIR 2800–6371/33–70, Coronal T1-weighted 100–1610/10–12, Axial STIR 4000–9830/32–65, Axial T2-weighted fast suppressed 2500–13177/71–105, Coronal HASTE 900/82–86, Axial HASTE 900/79–85, Sagittal HASTE 900/83–87 3 T Coronal STIR 2900–5050/30–41, Coronal T1-weighted 480–820/9–10, Axial STIR 2500–9860/27–38, Axial T2 weighted fast suppressed 4240–17264/77–113, Coronal HASTE 1000/83–87, Axial HASTE 1230/87, Sagittal HASTE 1000/87	1.5 T Coronal STIR 4–6.6, Coronal T1-weighted 4–6.6, Axial STIR 4–8, Axial T2-weighted fast suppressed 4–8, Coronal HASTE 6–7, Axial HASTE 8–10, Sagittal HASTE 7–10 3 T Coronal STIR 4–5.5, Coronal T1-weighted 4–5.5, Axial STIR 4–7, Axial T2-weighted fast suppressed 4–7, Coronal HASTE 8–20, Axial HASTE 8, Sagittal HASTE 8.8	1.5 T Coronal STIR 256 × 256, Coronal T1-weighted 320 × 256, Axial STIR 256 × 256, Axial T2-weighted fast suppressed 320 × 320, Coronal HASTE 256 × 243, Axial HASTE 256 × 243, Sagittal HASTE 256 × 243 3 T Coronal STIR 256 × 256, Coronal T1-weighted 384 × 288, Axial STIR 256 × 256, Axial T2-weighted fast suppressed 320 × 320, Coronal HASTE 256 × 256, Axial HASTE 256 × 256, Sagittal HASTE 256 × 192	No
Saya [33]	/	Yes	Two independent radiologist with at least 5 years’ experience	Axial T1-weighted gradient echo, axial fat-suppressed T2-weighted HASTE, axial DWIBS whole body, coronal T1-weighted VIBE DIXON	1.5 T MRI machine (Siemens, Erlangen, Germany)	1.5 T	38–40 cm	Axial T1-weighted gradient echo 247/4.36, axial fat-suppressed T2-weighted HASTE 1000/84, axial DWIBS whole body 8600/72, coronal T1-weighted VIBE DIXON 6.97/2.39	Axial T1-weighted gradient echo 8, axial fat-suppressed T2-weighted HASTE 8, axial DWIBS whole-body 8, coronal T1-weighted VIBE DIXON 5	Axial T1 weighted gradient echo 182 × 320, axial fat-suppressed T2-weighted HASTE 208 × 256, axial DWIBS whole-body 128 × 128, coronal T1-weighted VIBE DIXON 192 × 192	No
Bojadzieva [13]	/	Yes	All scans reviewed by primary author, lesions reviewed by diagnostic radiologist	WB-MRI—Scout, DWI, T2 TIRM, T1FS Post VIBE, T1FS Post. Brain MRI—DWI, T2, FLAIR, T2, T1, T1 Post	WB-MRI—Siemens Aera 1.5T, brain MRI—GE SIgna HD 1.5T, Siemens Aera 1.5T	1.5T	WB-MRI—([45 cm × 45 cm for Scout, DWI, T2 TIRM], [24 cm × 24 cm for T1FS post VIBE head, 44cm × 44 cm for T1FS post VIBE chest to pelvis, 38cm × 38 cm for T1FS post VIBE thighs to toes], [34cm × 34 cm for T1FS post]). Brain MRI—22 cm for all sequence except for 24 cm for T1 post sagittal view.	WB-MRI TR—1500, 8300, 5130 (H&N) 3000 (ch/ab) 5500 (pel-toes), 12, 4.09 (ch) 4.38 (ab) 4.51 (pel), 4.51, <500 for Scout, DWI, T2 TIRM, T1FS Post VIBE, T1FS Post, respectively. Brain MRI TR- 8000, 3600–4400, 10000, 675–825, 500–600, 500–600 for DWI, T2, FLAIR, T2, T1, T1 Post, respectively	WBMRI—6, 5, 6 (chest/ab) 5 (others), 5, 5, 5, 4 for Scout, DWI, T2 TIRM, T1FS Post VIBE, T1FS Post, respectively. Brain MRI—5 for all phases.	WBMRI—320 × 256 160 × 108 384 × 207 256 × 192 384 × 171 288 × 132 320 × 256 for Scout DWI T2 TIRM T1FS Post VIBE (ch, abd, pelv) T1FS Post, respectively. Brain MRI—128 × 128 320 × 224 256 × 192 256 × 192 256 × 192 256 × 192 256 × 192 256 × 192 for DWI, T2, FLAIR T2, T T1 Post, respectively.	Yes
Mai [35]	45 min (mean)	No	/	Cor STIR, Cor 3D T1, Ax STIR	-	-	N/a	TR—3750, 6.36, 4990 for Cor STIR, Cor 3D T1, Ax STIR, respectively. TE—60, 4.77, 61 for Cor STIR, Cor 3D T1, Ax STIR, respectively	N/a	N/a	Yes
O’Neill [36]	60–90 min (range)	Yes	Neuroradiology for brain, body radiologist for body, study radiologist independently reviewed all scans for accurate representation	N/a	-	3T	N/a	N/a	N/a	N/a	No
Paixiao [25]	25–35 min (range)	Yes	Experienced radiologists	Coronal T1 weighted, short TI inversion recovery, axial diffusion weighted	Signa Excite HT	1.5T	N/a	N/a	N/a	N/a	No
Omran [12]	/	Yes	Two consultant radiologist	SSFSE/Haste Fat ST T2, DIXON T1, T2/EPI b50_400_800	1.5T Siemens	1.5T	400 × 275, 416/315, 420/338	1000/82, 165/60, 5600/60	Five for all three phases	256/123, 256/134, 192/156	No
Kagami [34]	/	No	/	Adult—T2-weighted imaging with and without fat suppression using HASTE technique, whole-body diffusion-weighted imaging (DWI), and pre and post-T1 weighted imaging using DIXON technique. Children—short tau inversion recovery (STIR) or T2-weighted fat sup-pressed images and T1-weighted images.	1.5T scanner (Aera, Sola, and Espree, Siemens Healthcare) for adults, WB-MRI examinations were per-formed on a 1.5-Tesla or a 3.0-Tesla scanner (Siemens Health) for children	1.5T (adult), 1.5T or 3T (children)	N/a	N/a	N/a	N/a	No

WB-MRI: whole body MRI, ST: slice thickness, ET: echo time, RT: repetition time, FOV: field of view.

**Table 3:** Incidence of new cancer and critical findings.

Author	Patients with Subsequent Cancer on MRI		Location of Lesion (Multiple Lesions Found in Some Patients)										Critical Findings
			Abdomen	Pelvis	Chest	Head	Neck	LL	UL	Spine	Breast	Other	
	No.	%											
Anupindi [20]	1	4	0	0	0	0	1	0	0	0	0	0	Sensitivity of WB-MRI—100% Specificity of WB-MRI—94% PPV of WB-MRI—25% NPV of WB-MRI—100%
Saya [33]	6	13.6	2	1	1	1	0	1	0	0	0	1	6/44 (13.6, 95% CI 5.2–27.4%) of the participants were diagnosed with cancer during the study.
Bojadzieva [13]	6	9.5	3	0	0	0	2	0	1	0	0	0	Screening WB-WRI in 6/63 (9.5%) of patients supports the inclusion of WB-MRI and brain MRI in the clinical management of individuals with LFS.
Mai [35]	5	4.3	0	0	4	1	0	0	0	0	0	0	Abnormal MRI findings requiring additional follow-up were identified in 32/116 WB-MRIs (27.5%). In total, 27/32 (84.4%) of the abnormal WB-MRIs required follow-up, including two site-specific biopsies, with results showing benign or normal findings.
O’Neill [36]	0	0	0	0	0	0	0	0	0	0	0	0	In total, 89% patients returned for second examinations (95% CI—67–99%). Considered successful feasibility study as upper bound of 95% Confidence Interval of success is ≥90%.
Paixiao [25]	3	5	2	1	0	0	0	0	1	0	0	0	1st round of WB-MRI—positive rate 11.8%, recall rate 11.8%, invasive investigation rate 3.4%, cancer detection rate 3.4%, execution success 95%. 2nd round of WB-MRI—positive rate 6.7%, recall rate 6.7%, cancer detection rate 1.7%, success rate 100%
Omran [12]	3	5	4	0	4	0	1	0	0	0	0	0	In total, 30 new lesions were identified by WB-MRI in 19 individuals (31%) requiring follow up; 9 of the 30 were malignant, in 3 patients. One was recurrence, one was new disease, and one was disseminated disease. All were asymptomatic
Kagami [34]	12	10.2	5	2	1	2	0	2	0	0	1	0	The most frequent intervention recommended after initial screening was short-term follow-up imaging (adults, 81%,pediatric, 60%), followed by immediate imaging (adults, 19%, paediatric, 40%). None of the adult cohort and one child in the paediatric cohort were recommended for invasive interventions after the initial screening, while three adults (3/68, 4.4%) and six children (6/50, 12%) were recommended for invasive interventions after subsequent screening.

WB-MRI: whole-body MRI, LL: lower limb(s), UL: upper limb(s).

**Table 4:** Cancer location and timing of MRI.

Author	Cancer Location		Initial/Subsequent MRI	
Omran [12]	1—Pleura, cervical LN 2—Mediastinal LN, pleura, liver, intraabdominal LN 3—Liver, intra-abdominal LN		1—Initial WBI MRI 2—Initial WB-MRI 3—Initial WB-MRI	
Paixiao [25]	1—Renal cell carcinoma (right and left kidney) 2—Grade 1 chondrosarcoma (left SI joint) 3—High-grade sarcoma with muscle differentiation (right humerus)		1—Initial WBI MRI 2—Initial WB-MRI 3—Subsequent WB-MRI	
O’Neill [36]	No new cancers identified		-	
Mai [35]	1—Lung adenocarcinoma 2—Lung adenocarcinoma 3—Intermediate grade sarcoma in left 4th rib 4—Low-grade spindle cell sarcoma on left chest skin biopsy 5—Brain cancer—astrocytoma frontal lobe		Not specified	
Kagami [34]	Adults 1—RCC 2× primaries 2—Infiltrating ductal carcinoma 3—Lung adenocarcinoma 4—Endocervical adenocarcinoma 5—Leiomyosarcoma	Paediatric 1—Colorectal adenocarcinoma 2—Rhabdomyosarcoma 3—Atypical astrocytoma 4—Colonic adenocarcinoma 5—Pancreatic NET 6—Osteosarcoma 7—High-grade astrocytoma	Adults 1—Initial WB-MRI 2—Subsequent WB-MRI 3—Subsequent WB-MRI 4—Subsequent WB-MRI 5—Initial WB-MRI	Paediatric 1—Subsequent WB-MRI 2—Initial WB-MRI 3—Subsequent WB-MRI 4—Subsequent WB-MRI 5—Subsequent WB-MRI 6—Subsequent WB-MRI 7—Initial WB-MRI
Bojadzieva [13]	1—Recurrent soft tissue sarcoma, new primary abdominal soft tissue sarcoma 2—Sarcoma metastasis 3—Papillary thyroid cancer 4—Gastric cancer 5—Bilateral thyroid cysts not significant on WB-MRI, latera shown to be thyroid cancer 6—Shoulder lesion, excision showed liposarcoma		Not specified	
Saya [33]	1—Low-grade astrocytoma 2—Low-grade myxosarcoma in abdominal wall 3—Renal cell carcinoma, leiomyosarcoma 4—Chondroblastic osteosarcoma 5—Pericardial cyst on WB-MRI—diagnosed as mediastinal sarcoma 6—B cell acute lymphoma		1—Initial WB-MRI 2—Initial WB-MRI 3—Initial WB-MRI 4—Initial WB-MRI 5—Initial WB-MRI 6—Initial WB-MRI	
Anupindi [20]	1—Papillary thyroid carcinoma		1—Initial WB-MRI	

LN: lymph node, RCC: renal cell carcinoma, WB-MRI: whole-body MRI, SI: sacro-iliac.

## Data Availability

Data sharing is not applicable.

## Supplementary Materials

The following supporting information can be downloaded at: https://www.mdpi.com/article/10.3390/jcm13051223/s1. Supplementary Material S1: Search strategy. Supplementary Material S2: Risk of Bias assessment (Newcastle–Ottawa scale).
