# Supplementary material for: Whole-Body MRI Screening for Carriers of Germline TP53 Mutations—A Systematic Review and Meta-Analysis
Source: J Clin Med. 2024 Feb 21;13(5):1223. doi: 10.3390/jcm13051223 (PMC10931931; doi:10.3390/jcm13051223)
Supplement: Supplementary file 1 [file jcm-13-01223-s001.zip › jcm-2863329-supplementary.pdf]

(Whole body OR wb OR screen*) AND (MRI or Magnetic Resonance) AND (Li Fraumeni OR p53)

Supplementary Material S1: Search strategy

Author	Selection				Comparability	Outcome			Quality
	**Representativeness of the exposed cohort**	**Sample size (<25 = no star)**	**Non-respondents**	**Ascertainment of the exposure**	**The subjects in different outcome groups are comparable**	**Assessment of outcome**	**Statistical test**	**Period (<4weeks)**	
Anupindi	✸	✸	/	✸	✸	/	✸	/	5
Saya	✸	✸	/	✸	✸	✸	✸	✸	7
Bojadzieva	✸	✸	/	/	✸	✸	/	✸	5
Kagami	✸	✸	/	/	✸	✸	/	✸	5
Mai	✸	✸	✸	/	✸	/	✸	✸	6
O'Neill	✸	✸	✸	/	✸	/	✸	✸	6
Omran	✸	✸	✸	/	✸	/	✸	/	5
Paixiao	✸	✸	/	/	✸	/✸	✸	/	5

Supplementary Material S2: Risk of bias assessment (Newcastle-Ottawa scale)
